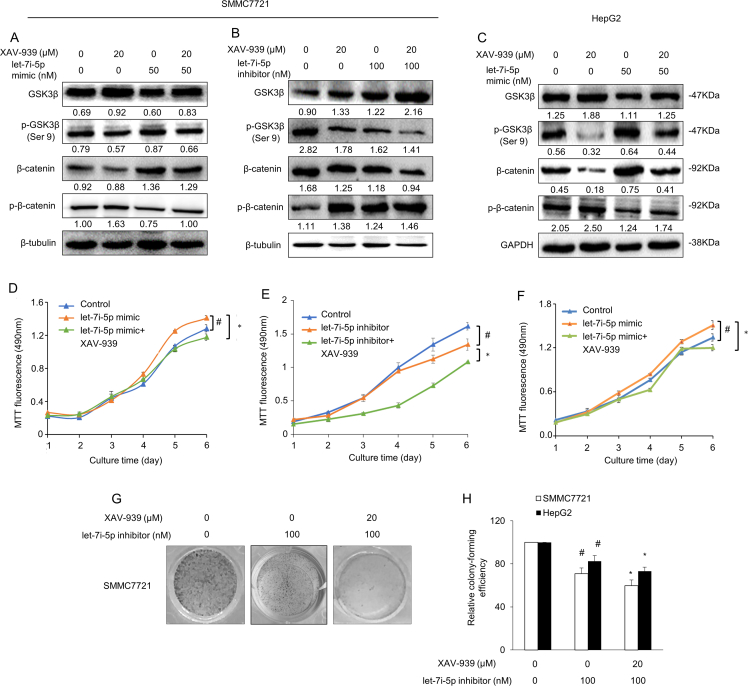# Let-7i-5p maintains the stemness via R-spondin2/Wnt pathway in hepatocellular carcinoma

**DOI:** 10.1016/j.gendis.2023.101096

**Published:** 2023-09-14

**Authors:** Qinghua Wei, Xueshan Dai, Jiahui Wei, Wenwei Sun, Xiaoqian Yang, Yi Ding, Yuxin Zhang, Xin Guo, Yi Chen

**Affiliations:** a College of Pharmaceutical Sciences & Chinese Medicine, Southwest University, Chongqing 400716, China; b Center for Basic Medical Research, TEDA International Cardiovascular Hospital, Chinese Academy of Medical Sciences & Peking Union Medical College, Tianjin 300457, China; c Chongqing Key Laboratory of New Drug Screening from Traditional Chinese Medicine, Chongqing 400716, China; d Pharmacology of Chinese Materia Medica – the Key Discipline Constructed by the State Administration of Traditional Chinese Medicine, Chongqing 400716, China; e Division of Cell, Developmental and Integrative Biology, School of Medicine, South China University of Technology, Guangzhou, Guangdong 510006, China; f National Demonstration Center for Experimental Pharmacy Education (Southwest University), Chongqing 400716, China; g Engineering Research Center of Coptis Development and Utilization (Ministry of Education), College of Pharmaceutical Sciences and Chinese Medicine, Southwest University, Chongqing 400716, China

Cancer stem cells (CSCs) are related to tumorigenesis, recurrence, metastasis, and drug resistance in hepatocellular carcinoma (HCC).^1^ Let-7i is a noncoding small RNA, belonging to the famous miRNA let-7 family. Recently, let-7i has been revealed as a tumor suppressor on CSCs in ovarian cancer.^2^ However, let-7i is overexpressed in side population cells of rat HCC.^3^ It is unknown about the role of let-7i on human CSCs in HCC. Wnt pathway has critical roles in tumorigenesis, including differentiation, proliferation, and drug resistance of liver cancer. Let-7i inhibits osteogenesis by targeting Wnt pathway.^4^ Moreover, let-7i-3p suppresses cell growth and β-catenin in hepatoblastoma.^5^ Nevertheless, the effect of let-7i-5p on Wnt pathway needs to be uncovered in HCC. In this study, we aimed to investigate the effect and mechanism of let-7i-5p on stemness maintenance of CSCs in HCC.

Spheres display stem cell properties in many kinds of cancers. Culturing in serum-free suspension for 7–10 d, HCC spheres of SMMC7721 and HepG2 cells were round and ovoid with strong refractive properties (Fig. S1A). The let-7i-5p level increased in spheres versus adherent cells of SMMC7721 and HepG2 cells (Fig. 1A). Both HCC cell lines were then transfected with let-7i-5p mimic or let-7i-5p inhibitor. The expression of let-7i-5p increased by more than three folds using let-7i-5p mimic, which was reduced using let-7i-5p inhibitor (Fig. S1B, C). After transfection, the stemness maintenance of CSCs in HCC was observed, including CSC markers (CD133 and EpCAM), stem-cell genes, sphere formation, plate cloning, soft agar cloning, and cell growth. The protein levels of CD133 and EpCAM were raised in the let-7i-5p mimic group, while let-7i-5p inhibitor suppressed CD133 and EpCAM proteins (Fig. 1B; Fig. S1D–G). In RT-qPCR assay, let-7i-5p mimic promoted the expression of stem-cell genes, such as SOX2, Oct4, Nanog, and Bmi1. Meanwhile, let-7i-5p inhibitor caused the down-regulation of these four stem-cell genes (Fig. S1H–K). The larger, tighter, and more spheres were found in the let-7i-5p mimic group. However, when let-7i-5p decreased, spheres became smaller, looser, and less compared with the control (Fig. 1C; Fig. S1L–N). Especially using let-7i-5p inhibitor, few spheres formed in SMMC7721 cells. In the cell growth assay, the up-regulation of let-7i-5p accelerated the proliferation rate. The down-regulation of let-7i-5p appeared the opposite effects on day 6 (Fig. 1D; Fig. S2A, B). In the plate colony formation assay, the volume and number of clones were enlarged by let-7i-5p mimic, however, let-7i-5p inhibitor embraced the opposite effects (Fig. 1E; Fig. S2C–E). Similarly, let-7i-5p affected the formation of soft agar clones (Fig. S2F–I).

**Figure 1:**
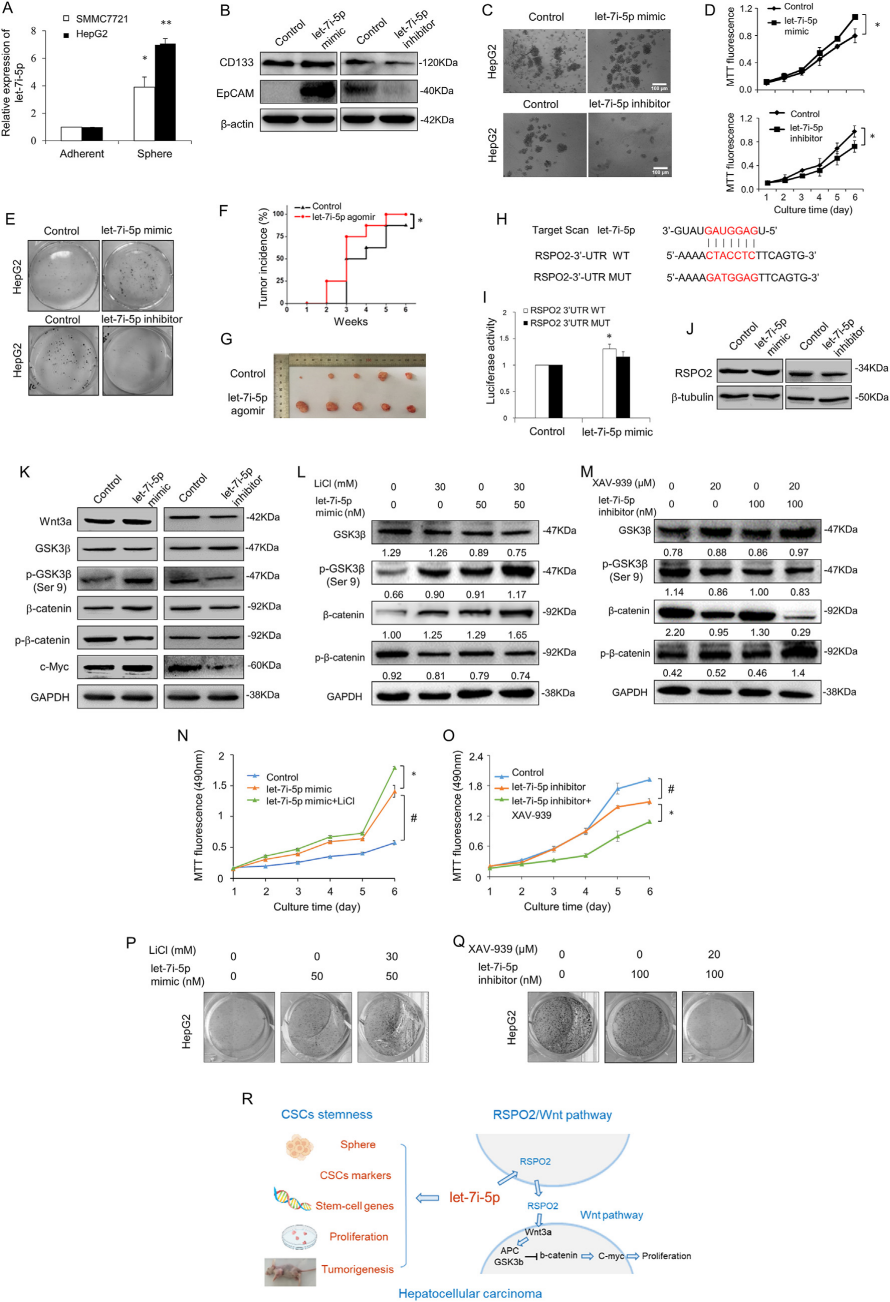
Let-7i-5p maintains the stemness via R-spondin2/Wnt pathway in hepatocellular carcinoma. **(A)** After being cultured for 7–10 d, HCC spheres of SMMC7721 and HepG2 cells were collected for RT-qPCR analysis of let-7i-5p. **(B)** In Western blot assay, let-7i-5p mimic or inhibitor regulated the CSC markers (CD133 and EpCAM) in HepG2 cells. **(C)** Sphere formation detection of HepG2 cells using let-7i-5p mimic or inhibitor. Upper and lower scale bars = 100 μm. **(D)** The growth of HepG2 HCC cells was tested for 6 d by MTT assay. **(E)** The plate colony formation was cultured for 7–10 d. **(F)** After being transfected with 100 nM let-7i-5p agomir for 24 h, HepG2 cells were injected into the hypodermis of nude mice for 6 weeks. The tumor incidence was detected once per week. **(G)** The appearance and volume of tumor tissue were detected. **(H)** The binding sequence was predicted and designed for the mutant RSPO2 3'-UTR plasmid. **(I)** In the dual-luciferase reporter assay, luciferase activities were measured after co-transfected let-7i-5p mimic with wild-type or mutant RSPO2 3'-UTR plasmids in HepG2 cells, respectively. **(J)** The protein expression of RSPO2 was tested by Western blot assay. **(K)** After transfection, the protein expression of Wnt pathway was observed in HepG2 cells. **(L)** Let-7i-5p mimic cooperated with LiCl to up-regulate the Wnt pathway. **(M)** The protein levels of Wnt pathway were inspected using let-7i-5p inhibitor and XAV-939 in HepG2 cells. **(N, O)** The cell growth of HepG2 cells was detected during 6 d. **(P, Q)** Observation of the plate colony formation after treatment with let-7i-5p mimic and LiCl or let-7i-5p inhibitor and XAV-939. **(R)** The schematic shows that let-7i-5p can maintain the stemness of HCC cells. Let-7i-5p accelerates the proliferation by targeting RSPO2 and activating Wnt pathway. ^∗^*P* < 0.05 and ^∗∗^*P* < 0.01 versus the control. Columns, mean (F, G, *n* = 5; A–E, I–K, N–Q, *n* = 3); bars, standard deviation; HCC, hepatocellular carcinoma; CSCs, cancer stem cells.

CSCs are considered one of the major factors of tumorigenesis *in vivo*. So, we elucidated the influence of let-7i-5p on tumorigenesis. After being transfected with let-7i-5p agomir, HepG2 cells were injected into nude mice. During 6 weeks, let-7i-5p agomir facilitated tumor growth (Fig. 1F). The tumor volume and weight of let-7i-5p agomir group were larger and heavier than those of the control group at 6 weeks (Fig. 1G; Figs. S3A–C). At the same time, let-7i-5p agomir induced the levels of let-7i-5p, CD133, and EpCAM protein in tumor tissues (Figs. S3D–F). Collectively, let-7i-5p maintained the characteristics of HCC CSCs *in vitro* and *in vivo*.

Next, we investigated the potential target gene of let-7i-5p. Using microRNA databases, R-spondin2 (RSPO2) was predicted to be a putative target of let-7i-5p. According to the potential binding sites, the mutant plasmid was constructed with the partial mutation of RSPO2 3'-UTR sequences (Fig. 1H). In dual-luciferase reporter assay, let-7i-5p mimic enhanced the luciferase activities of wild type without mutant RSPO2 in both cell lines (Fig. 1I; Fig. S4A). Moreover, RSPO2 gene was amplified by let-7i-5p mimic, and diminished by let-7i-5p inhibitor (Fig. S4B, C). As a result, let-7i-5p expressed the effect on RSPO2 protein resembling those on RSPO2 gene (Fig. 1J; Fig. S4D–F). Therefore, it indicated that RSPO2 was the direct target of let-7i-5p.

RSPO2, as an activator of Wnt pathway, can induce proliferation. Previously, let-7i-5p improved the proliferation. Meanwhile, RSPO2 was proved as one of the target genes of let-7i-5p. It is unclear whether the mechanism of let-7i-5p on proliferation is related to RSPO2/Wnt pathway. Then, the regulation of let-7i-5p on Wnt pathway was tested. Let-7i-5p mimic promoted the mRNA levels of Wnt3a, β-catenin, and c-Myc, accompanied by descending mRNA levels of APC and GSK3β (Fig. S5A, B). The result was reversed after transfection with let-7i-5p inhibitor (Fig. S5C, D). Furthermore, let-7i-5p mimic enhanced Wnt3a protein. Then the down-stream proteins were activated, including leading GSK3β to p-GSK3β (Ser9), inhibiting β-catenin to p-β-catenin, and increasing c-Myc (Fig. 1K; Fig. S5E, F, I). Meanwhile, let-7i-5p inhibitor showed the contrary effect on the proteins of Wnt pathway (Fig. 1K; Fig. S5G, H, J). In conclusion, let-7i-5p could markedly activate Wnt pathway.

LiCl is the special agonist of Wnt pathway. After being treated with 30 mM LiCl, Wnt pathway was activated. When being treated with LiCl and let-7i-5p mimic, the activation of Wnt pathway was intensified by depressing GSK3β and p-β-catenin and rising p-GSK3β (Ser9) and β-catenin (Fig. 1L; Fig. S6A). However, let-7i-5p inhibitor prevented the activating Wnt pathway of LiCl (Fig. S6B, C). LiCl and let-7i-5p mimic presented the synergistic promotion of cell growth and clone ability (Fig. 1N, P; Fig. S6D, G, H). On the contrary, LiCl and let-7i-5p inhibitor showed an adverse influence on cell growth (Fig. S6E, F).

XAV-939 has a widespread availability as Wnt pathway inhibitor. After being treated with 20 μM XAV-939, the decline of Wnt pathway activity was found. When being treated with XAV-939 and let-7i-5p mimic, the down-regulating Wnt pathway was reversed (Fig. S7A, C). However, let-7i-5p inhibitor caused the synergistic inhibitory with XAV-939 on Wnt pathway (Fig. 1M; Fig. S7B). As a result, XAV-939 slowed down the cell growth accelerated by let-7i-5p mimic (Fig. S7D, F). After being treated with XAV-939, the proliferative suppression of let-7i-5p inhibitor was dramatically intensified (Fig. 1O, Q; Fig. S7E, G, H). These data indicated that let-7i-5p possessed the synergistic effect with LiCl and the antagonistic effect with XAV-939 on Wnt pathway and proliferation.

In brief, let-7i-5p regulated the CSC stemness maintenance of HCC. Simultaneously let-7i-5p ameliorated the proliferation by targeting RSPO2 and activating Wnt pathway (Fig. 1R). These findings illuminate the intrinsic mechanism of let-7i-5p in liver CSCs and supply potential novel therapeutic strategies for HCC.

## Author contributions

Qinghua Wei, Xueshan Dai, Jiahui Wei, and Wenwei Sun: data curation, investigation, methodology, project administration, validation, and roles/writing-original draft. Xiaoqian Yang, Yi Ding, and Yuxin Zhang: data curation, investigation, and methodology. Xin Guo and Yi Chen: conceptualization, data curation, funding acquisition, methodology, supervision, roles/writing-original draft, and writing-review & editing.

## Conflict of interests

The authors declare that they have no competing interests.

## Funding

This work was supported by the National Natural Science Foundation of China (No. 81773984, 81402441), Chongqing Natural Science Foundation of China (No. CSTC2020JCYJ-MSXMX0451), Traditional Chinese medicine research project of Chongqing Health Bureau (No. 2020ZY023665), Chinese Medicine Rehabilitation – the Key Discipline Constructed by Chongqing Health Bureau (No. 2021-4322190044), Fundamental Research Funds for the Central Universities (China) (No. XDJK2012C054), and Southwest University Doctor Foundation (Chongqing, China) (No. SWU111074).

## Figures and Tables

**figs1:**
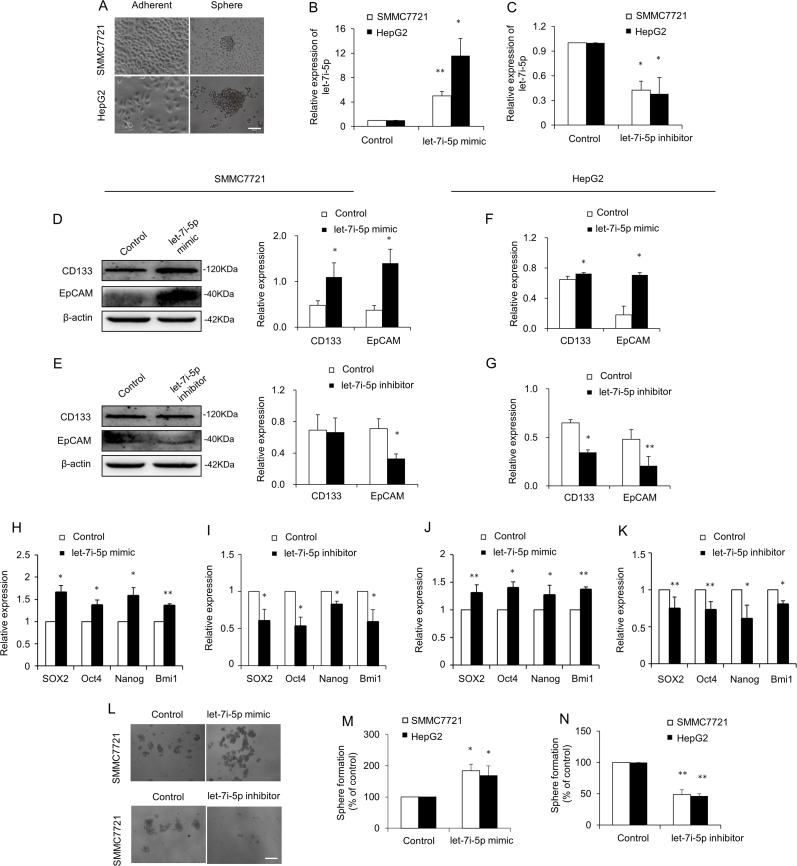


**figs2:**
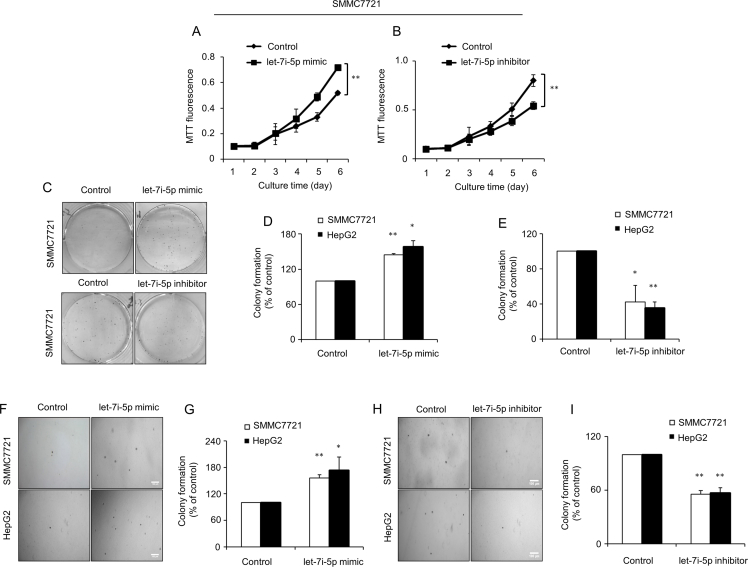


**figs3:**
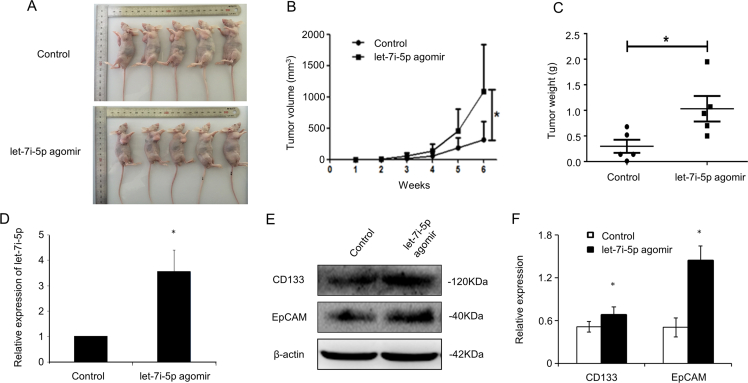


**figs4:**
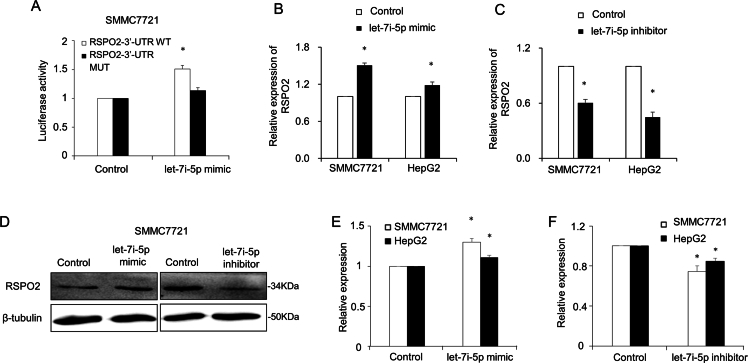


**figs5:**
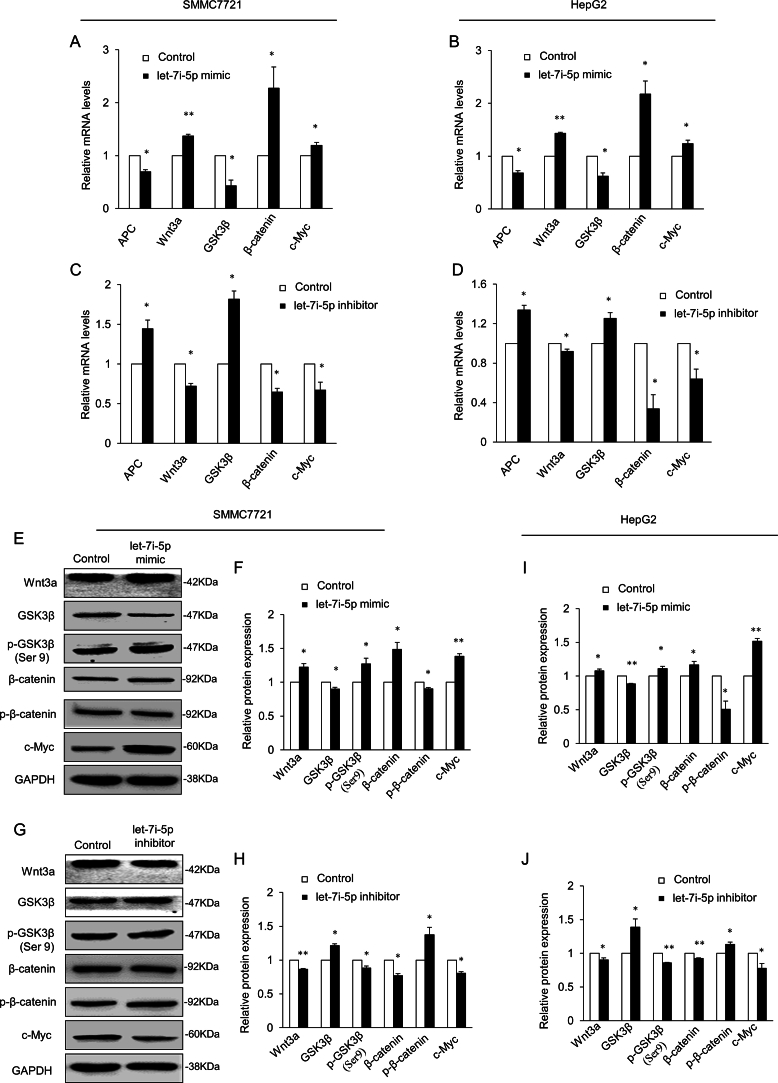


**figs6:**
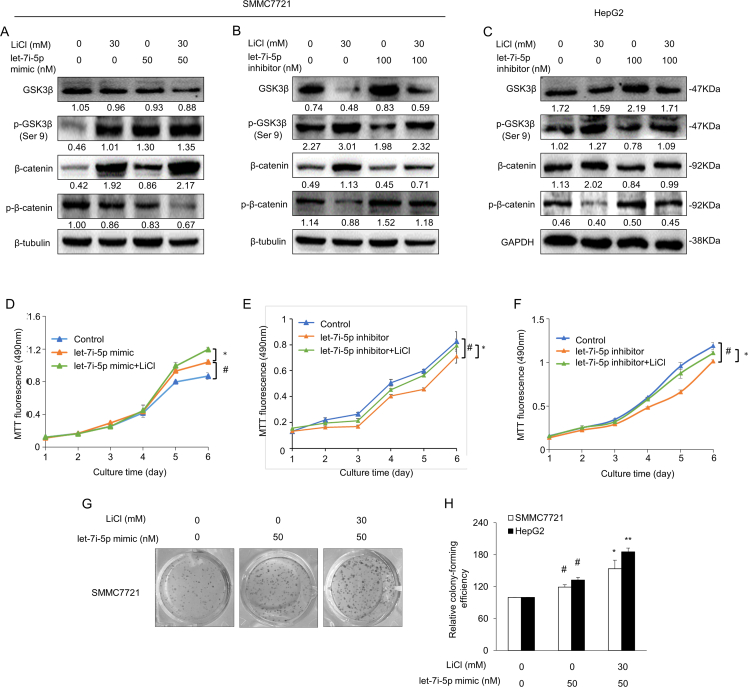


**figs7:**