# Supplementary material for: Let-7i-5p maintains the stemness via R-spondin2/Wnt pathway in hepatocellular carcinoma
Source: Genes Dis. 2023 Sep 14;11(4):101096. doi: 10.1016/j.gendis.2023.101096 (PMC10906152; doi:10.1016/j.gendis.2023.101096)
Supplement: Multimedia component 1 [file mmc1.docx]

**Materials and methods**

**Animals and drugs**

The 16-18 g male nude mice were purchased from Beijing Huafukang biotechnology Co. Ltd (Certification No: SCXK [jing] 2014-0004). They were raised at *T*a of 20±2℃ and 12 h light/dark cycle, free access to food and water in the Experimental Center, College of Pharmaceutical Sciences, Southwest University. This study was strictly carried out with the recommendations of Laboratory Animals of Southwest University (Approval No. 0002183). Anesthesia and other necessary methods were provided to reduce suffering.

LiCl (purity > 99%, Sigma-Aldrich, USA) was dissolved in PBS. XAV-939 (purity > 99%, Med ChemExpress, USA) was dissolved in DMSO. Both of them were stored at -20℃ away from light.

**Cell culture and** [**transfection**](javascript:;)

The SMMC7721 and HepG2 cell lines were purchased from the Cell Bank at the Shanghai Institutes of Chinese Sciences Academy. Both cells were cultured in RPMI 1640 and maintained at 37℃ with 5% CO_2_. The cells were transfected with 50 nM let-7i-5p mimic, 100 nM let-7i-5p inhibitor, 100 nM let-7i-5p agomir, or their respective controls (Ruibo biotechnology, China) for 24 h by Lipofectamine^TM^ 2000 (Invitrogen, USA) according to the manufacturer’s protocol. Opti-MEM (Gibco, USA) medium was replaced after 4-6 h of transfection.

**Sphere formation assay**

The sphere medium was prepared with Mammocult^TM^ Human Medium Kit (Stem cell, USA), adding hydrocortisone (0.48 μg·ml^−1^, Stem cell, USA) and heparin (4 μg·ml^−1^, Stem cell, USA). 7-10×10^3^ HCC cells/well were cultivated with sphere medium in 24-well ultra-low adhesion plates (Corning, USA) for 7-10 d. Then HCC spheres were observed and calculated under microscope.

**Plate and soft agar colony formation assay**

Briefly, as described previously^2^, 5-8×10^2^ transfected cells/well were seeded in 6-well plates for plate colony formation. In brief, 5-8×10^2^ cells, 1.2% agar solution, and 2×RPMI1640 medium containing 20% FBS were mixed (1:1:1), and added to 6-well plates for soft agar colony formation. After 7-10 d, plate or soft agar clones (>50 cells) were counted and imaged under the microscope.

**Cell growth assay**

8-10×10^2^ transfected cells were inoculated in 96-well plates. From 1 to 6 d, the cell growth was measured with MTT method.

**Subcutaneous tumorigenesis in nude mice**

1×10^6^ HepG2 cells, transfected with 100 nM let-7i-5p agomir, were mixed with Matrigel (BD Biosciences, CA) (1:1), then injected into the hypodermis of nude mice. Body weight, tumor growth and volume were detected twice a week for 6 weeks. Tumor volume was calculated using formula, V=1/2 (width^2^×length).

**Dual-Luciferase reporter assay**

Let-7i-5p mimic was co-transfected with 200 ng wild-type or mutant RSPO2 3’-UTR plasmids (Ruibo biotechnology, China) respectively for 48 h. Firefly and Renilla luciferase activities were tested following the instruction of Dual-Luciferase reporter assay (Promega, USA). Firefly luciferase was used as internal parameter for normalization.

**RNA isolation and RT-qPCR**

Briefly, after mRNA extraction with TRIzol reagent (Invitrogen, USA), PrimeScript^TM^ RT reagent Kit (Takara, Japan) was used for cDNA synthesis. SYBR^TM^ Mix (Thermo Fisher Scientific, USA) was performed with RT-qPCR primers (Invitrogen, China, Table S1) using 2^-△△CT^ method normalized to GAPDH. The stem-loop RT primers of let-7i-5p and U6 were produced by Ruibo biotechnology, China.

**Western blot**

Briefly, the proteins of cells and tissues were lysed and extracted by RIPA (Dingguo Changsheng, China), and transferred to PVDF membrane (Millipore, USA). Proteins were bound to the primary antibodies and horseradish peroxidase-linked secondary antibodies (Table S2), then [visualiz](javascript:;)ed in Tanon 5200 system (Tanon, China). GAPDH, β-actin or β-tubulin was used as the normalization control for protein quantification.

**Statistical analysis**

Statistical significance was assessed by comparing mean ± *SD* values, using Student’s *t* tests for independent groups, and One-way ANOVA analysis for multiple groups. The tumor incidence was analyzed by Kaplan-Meyer method. Statistical analysis was performed by SPSS 21.0 software. *P*<0.05 was considered statistically significant.

Table S1 Primer sequence

| Gene |  | Sequence |
| --- | --- | --- |
| Sox2 | Forward | 5’-ACACCAATCCCATC CACACT-3’ |
|  | Reverse | 5’-GCAAACTTCCTGCAAAGCTC-3’ |
| Otc4 | Forward | 5’-GTTCTTCATTCACTAAGGAAGG-3’ |
|  | Reverse | 5’-CAAGAGCATCATTGAACTTCAC-3’ |
| Nanog | Forward | 5’-CTCTCCTCTTCCTTCCTCCAT-3’ |
|  | Reverse | 5’-TTGCGACACTCTTCTCTGC-3’ |
| Bmi-1 | Forward | 5’-TGGATCGGAAAGTAAACAAAGAC-3’ |
|  | Reverse | 5’-TGCATCACAGTCATTGCTGCT-3’ |
| RSPO2 | Forward | 5’-ACAATACTGTGTCCAACCAT-3’ |
|  | Reverse | 5’-TCCTCTTCTCCTTCGCCTTT-3’ |
| Wnt3a | Forward | 5’-GCCAGCCACATGCACCTCAA-3’ |
|  | Reverse | 5’-GCGACCACCAGCATGTCTTCAC-3’ |
| APC | Forward | 5’-AGGAGTTTAGCATGGCCCTT-3’ |
|  | Reverse | 5’-AGAATCGCTTGAACCTGGGA-3’ |
| GSK3β | Forward | 5’-GGAAGGTTGAGATGGGTGGA-3’ |
|  | Reverse | 5’-TCCAAACGATTCTCCTGCCT-3’ |
| β-catenin | Forward | 5’-GCTTCTCAAGGAGCTTACGC-3’ |
|  | Reverse | 5’-CATGTCACAGGTCGCTGATG-3’ |
| c-Myc | Forward | 5’-AACACACAACGTCTTGGAGC |
|  | Reverse | 5’-GCACAAGAGTTCCGTAGCTG |
| GAPDH | Forward | 5’-GACAGTCAGCCGCATCTTCT-3’ |
|  | Reverse | 5’-TTAAAAGCAGCCCTGGTGAC-3’ |

Table S2 Antibody information of western blot

| Antibody name | Dilution | Purchasing companies |
| --- | --- | --- |
| mouse anti-CD133 | 1:200 | Miltenyi, Germany |
| mouse anti-EpCAM | 1:500 | Cell Signaling Technology, USA |
| goat-anti-RSPO2 | 1:1000 | R&D System, USA |
| rabbit anti-Wnt3a | 1:1000 | Cell Signaling Technology, USA |
| rabbit anti-GSK3β | 1:1500 | Wanleibio, China |
| rabbit anti-p-GSK3β (Ser9) | 1:1000 | Wanleibio, China |
| rabbit anti-β-catenin | 1:1000 | Bioss, China |
| rabbit anti-p-β-catenin | 1:1000 | Bioss, China |
| rabbit anti-c-Myc | 1:800 | Wanleibio, China |
| mouse anti-β-actin | 1:3000 | Sigma-Aldrich, USA |
| rabbit anti-β-tublin | 1:1000 | Wanleibio, China |
| rabbit anti-GAPDH | 1:1000 | Wanleibio, China |
| HRP-labeled rabbit anti-goat | 1:5000 | Multi sciences, China |
| HRP-labeled goat anti-rabbit | 1:5000 | Multi sciences, China |
| HRP-labeled goat anti-mouse | 1:3000 | Cell Signaling Technology, USA |

**Figure legends**

**Fig. S1. The stemness maintenance of HCC cells by let-7i-5p.** (A) After cultured for 7-10 d, HCC spheres of SMMC7721 and HepG2 cells were observed under microscope. (B, C) The let-7i-5p level was measured by RT-qPCR after the transfection of 50 nM let-7i-5p mimic or 100 nM let-7i-5p inhibitor for 24 h. (D, E) In western blot assay, let-7i-5p mimic or inhibitor regulated the CSCs markers (CD133 and EpCAM) in SMMC7721 cells. (F, G) The relative gray value statistics graphs of CD133 and EpCAM were represented the blots in Fig. 1B of HepG2 cells. (H-K) The expression of stem-cell genes (Nanog, SOX2, Oct4 and Bmi1) in HCC cells was analyzed by RT-qPCR. (L) Spheres of SMMC7721 cells were detected using let-7i-5p mimic or inhibitor. (M, N) Sphere formation efficiency of Fig. 1C and Fig. S1L was calculated based on the formula (spheres number ÷ number of seeded cells) × 100. ∗*P<* 0.05, ∗∗ *P<* 0.01 to Control. Columns, mean (n=3). Bars, SD. Upper and lower scale bar=100 μm. HCC, hepatocellular carcinoma; CSCs, cancer stem cells.

**Fig. S2. Let-7i-5p promoted the proliferation in HCC cells.** (A, B) The cell growth of SMMC7721 cells were tested for 6 d by MTT assay. (C) The plate colony of SMMC7721 cells were detected using let-7i-5p mimic or inhibitor. (D, E) Colony formation efficiency of Fig. 1E and Fig. S2C was calculated based on the formula (clones number ÷ number of seeded cells) × 100. (F-I) The measurement of soft agar colony formation after 7-10 d culture. ∗*P<* 0.05, ∗∗ *P<* 0.01 to Control. Columns, mean (n=3). Bars, SD. Upper and lower scale bar=100 μm. HCC, hepatocellular carcinoma.

**Fig. S3. Let-7i-5p facilitated the tumorigenesis of HepG2 cells *in vivo*.** (A) After transplantation for 6 weeks, nude mice were sacrificed for the observation of tumor tissue. (B, C) Detection of the volume and weight of tumor tissue. (D) The expression of let-7i-5p was observed by RT-qPCR in tumor tissue. (E, F) The proteins of CD133 and EpCAM, as the CSCs markers, were investigated by western blot assay. ∗*P<* 0.05, ∗∗ *P<* 0.01 to Control. Columns, mean (A-C, n=5; D-F, n=3). Bars, SD. HCC, hepatocellular carcinoma; CSCs, cancer stem cells.

**Fig. S4.** **RSPO2 as a target of let-7i-5p.** (A) Using Dual-Luciferase reporter assay, luciferase activities of SMMC7721 cells were measured after co-transfected let-7i-5p mimic with wild-type or mutant RSPO2 3’-UTR plasmids, respectively. (B, C) The mRNA level of RSPO2 was tested by RT-qPCR. (D) The proteins expression of RSPO2 was tested by western blot assay. (E, F) The relative gray value statistics graphs of RSPO2 were represented the blots in in Fig. 1J and Fig. S4D. ∗*P<* 0.05, ∗∗ *P<* 0.01 to Control. Columns, mean (n=3). Bars, SD. RSPO2, R-spondin2.

**Fig. S5. Let-7i-5p up-regulated the Wnt pathway.** (A-D) After transfection with let-7i-5p mimic or inhibitor for 24 h, the genes of Wnt pathway were investigated by RT-qPCR, including APC, Wnt3a, GSK3β, β-catenin, and c-Myc. (E-H) The protein levels of Wnt pathway were observed after increasing or decreasing let-7i-5p in SMMC7721 cells. (I, J) The relative gray value statistics graphs of Wnt pathway were represented the blots in Fig. 1K. ∗*P<* 0.05, ∗∗ *P<* 0.01 to Control. Columns, mean (n=3). Bars, SD.

**Fig. S6 Let-7i-5p augmented the proliferative activation of LiCl in HCC cells.** (A-C) Let-7i-5p mimic or inhibitor cooperated with LiCl to regulate Wnt pathway. The cell growth was detected during 6 d using let-7i-5p mimic or inhibitor with LiCl in SMMC7721 (D, E) and HepG2 (F) cells. (G, H) Observation of the plate colony formation after the transfection of let-7i-5p mimic and LiCl. # *P*< 0.05 to Control, ## *P*< 0.01 to Control, ∗ *P*< 0.05 to let-7i-5p mimic or inhibitor, ∗∗ *P*< 0.01 to let-7i-5p mimic or inhibitor. Columns, mean (n=3). Bars, SD. HCC, hepatocellular carcinoma.

**Fig. S7 The antagonistic interaction between let-7i-5p and XAV-939 on proliferation.** (A-C) The protein levels of Wnt pathway were inspected using let-7i-5p mimic or inhibitor with XAV-939. (D-F) After the treatments, the cell growth was observed for 6 d. (G, H) Decreasing plate colony appeared using let-7i-5p inhibitor and XAV-939. # *P*< 0.05 to Control, ## *P*< 0.01 to Control, ∗ *P*< 0.05 to let-7i-5p mimic or inhibitor, ∗∗ *P*< 0.01 to let-7i-5p mimic or inhibitor. Columns, mean (n=3). Bars, SD.
